# A prolonged methoxymorpholino doxorubicin (PNU-152243 or MMRDX) infusion schedule in patients with solid tumours: a phase 1 and pharmacokinetic study

**DOI:** 10.1054/bjoc.1999.0996

**Published:** 2000-02

**Authors:** E Fokkema, J Verweij, A T van Oosterom, D R A Uges, R Spinelli, O Valota, E G E de Vries, H J M Groen

**Affiliations:** Department of Pulmonology, Department of Pharmacy, Department of Internal Medicine, University Hospital Groningen, PO Box 30.001, Groningen, RB, 9700, The Netherlands; Rotterdam Cancer Institute and University Hospital, Rotterdam, The Netherlands; University Hospital Leuven, Belgium; Pharmacia & Upjohn, Milan, Italy

**Keywords:** methoxymorpholino doxorubicin, pharmacokinetics, phase I study

## Abstract

The aim of this phase I study was to assess feasibility, pharmacokinetics and toxicity of methoxymorpholino doxorubicin (MMRDX or PNU-152243) administered as a 3 h intravenous infusion once every 4 weeks. Fourteen patients with intrinsically anthracycline-resistant tumours received 37 cycles of MMRDX. The first cohort of patients was treated with 1 mg m^−2^of MMRDX. The next cohorts received 1.25 mg m^−2^and 1.5 mg m^−2^respectively. Common toxicity criteria (CTC) grade III/IV nausea and vomiting were observed in 1/18 cycles at 1.25 mg m^−2^and in 2/11 cycles at 1.5 mg m^−2^. Transient elevation in transaminases up to CTC grade III was observed in 2/16 cycles at 1.25 mg m^−2^and 4/11 cycles at 1.5 mg m^−2^. No cardiotoxicity was observed. At 1.25 mg m^−2^CTC grade IV neutropenia occurred in 1/17 cycles. At 1.5 mg m^−2^CTC grade III neutropenia was seen in 2/7 and grade IV in 3/7 evaluable cycles. Thrombocytopenia grade III was observed in 2/9 and grade IV in 1/9 evaluable cycles. One patient treated at 1.5 mg m^−2^died with neutropenic fever. Therefore, dose-limiting toxicity was reached and 1.25 mg m^−2^was considered the maximum tolerated dose for MMRDX as 3 h infusion. No tumour responses were observed. Pharmacokinetic parameters showed a rapid clearance of MMRDX from the circulation by an extensive tissue distribution. Renal excretion of the drug and its metabolite was negligible. In conclusion, prolongation of MMRDX infusion to 3 h does not improve the toxicity profile as compared with bolus administration. © 2000 Cancer Research Campaign


*British Journal of Cancer* (2000) **82**(4), 767–771
© 2000 Cancer Research Campaign
DOI: 10.1054/ bjoc.1999.0996, available online at http://www.idealibrary.com on IDEAL®

### A prolonged methoxymorpholino doxorubicin (PNU-152243 or MMRDX) infusion schedule in patients with solid tumours: a phase 1 and pharmacokinetic study

**E Fokkema[1], J Verweij[2], AT van Oosterom[3], DRA Uges[1], R Spinelli[4], O Valota[4], EGE de Vries[1] and HJM Groen[1]**

[1]Department of Pulmonology, Department of Pharmacy, Department of Internal Medicine, University Hospital Groningen, PO Box 30.001, 9700 RB Groningen, The Netherlands; [2]Rotterdam Cancer Institute and University Hospital, Rotterdam, The Netherlands; [3]University Hospital Leuven, Belgium; [4]Pharmacia & Upjohn, Milan, Italy

**Summary** The aim of this phase I study was to assess feasibility, pharmacokinetics and toxicity of methoxymorpholino doxorubicin (MMRDX or PNU-152243) administered as a 3 h intravenous infusion once every 4 weeks. Fourteen patients with intrinsically anthracycline-resistant tumours received 37 cycles of MMRDX. The first cohort of patients was treated with 1 mg $m^{-2}$ of MMRDX. The next cohorts received 1.25 mg $m^{-2}$ and 1.5 mg $m^{-2}$ respectively. Common toxicity criteria (CTC) grade III/IV nausea and vomiting were observed in 1/18 cycles at 1.25 mg $m^{-2}$ and in 2/11 cycles at 1.5 mg $m^{-2}$. Transient elevation in transaminases up to CTC grade III was observed in 2/16 cycles at 1.25 mg $m^{-2}$ and 4/11 cycles at 1.5 mg $m^{-2}$. No cardiotoxicity was observed. At 1.25 mg $m^{-2}$ CTC grade IV neutropenia occurred in 1/17 cycles. At 1.5 mg $m^{-2}$ CTC grade III neutropenia was seen in 2/7 and grade IV in 3/7 evaluable cycles. Thrombocytopenia grade III was observed in 2/9 and grade IV in 1/9 evaluable cycles. One patient treated at 1.5 mg $m^{-2}$ died with neutropenic fever. Therefore, dose-limiting toxicity was reached and 1.25 mg $m^{-2}$ was considered the maximum tolerated dose for MMRDX as 3 h infusion. No tumour responses were observed. Pharmacokinetic parameters showed a rapid clearance of MMRDX from the circulation by an extensive tissue distribution. Renal excretion of the drug and its metabolite was negligible. In conclusion, prolongation of MMRDX infusion to 3 h does not improve the toxicity profile as compared with bolus administration. © 2000 Cancer Research Campaign

**Keywords:** methoxymorpholino doxorubicin; pharmacokinetics; phase I study

Nowadays, anthracyclines play an important role in chemotherapeutic treatment of a wide variety of tumour types. The clinical use of anthracyclines is limited by their toxicity profile, especially irreversible, cumulative dose-related cardiotoxicity, and by intrinsic or acquired resistance of tumours cells. Mechanisms of this resistance consist of several pathways, such as overexpression of drug efflux pumps (i.e. P-glycoprotein), decrease in topoisomerase II enzyme levels, and increase in cellular detoxifying capacity (Kaye and Merry, 1985; Deffie et al, 1989; de Jong et al, 1990; Ford and Hait, 1990; Roninson, 1997).

Morpholinyl anthracyclines, such as methoxymorpholino doxorubicin (MMRDX or PNU-152243), were synthesized in search for new anthracyclines with at least partially novel modes of action, including activity on multidrug-resistant (MDR) tumours. MMRDX is a novel doxorubicin derivative in which the nitrogen atom of the daunosamine is enclosed in a methoxymorpholino ring (Figure 1). The drug easily fluxes into cells due to its high lipophilicity (Johnston and Glazer, 1983; Acton et al, 1984; Streeter et al, 1986). The working mechanism of morpholinyl anthracyclines appears to be different from other anthracyclines. Morpholinyl anthracyclines inhibit ribosomal gene transcription as well as topoisomerase I-mediated DNA-cleavage (Wassermann et al, 1988, 1990). MMRDX showed a potent cytotoxic activity in vitro on various tumour cell lines, including MDR tumour cell lines (Danesi et al, 1993; Kuhl et al, 1993; van der Graaf et al, 1995; Bakker et al, 1997). Metabolic conversion of MMRDX by human liver microsomes and NADPH potentiated the cytotoxicity in an ovarian carcinoma cell line (Lau et al, 1994). In animal studies, MMRDX showed activity against MDR xenografts (Ripamonti et al, 1992). However, in patients with various intrinsically anthracycline resistant solid tumours only a few tumour responses were observed (Vasey et al, 1995; Bakker et al, 1998). A phase I study (Vasey et al, 1995) with bolus injection of MMRDX, showed a maximum tolerated dose (MTD) at 1.5 mg $m^{-2}$ every 3 weeks and myelosuppression (neutropenia and thrombocytopenia) as dose-limiting toxicity (DLT). Nadirs occurred in the third week after treatment. A broad phase II study (Bakker et al, 1998) with 1.5 mg $m^{-2}$ MMRDX administered as bolus every 4 weeks also showed myelosuppression as major toxicity. Furthermore, both studies showed late and prolonged nausea, vomiting and transient elevations of hepatic transaminases. No signs of severe cardiotoxicity were observed in both human studies, although two patients had to be taken off study due to a decrease in left ventricular ejection fraction (LVEF) in the phase II study (Vasey et al, 1995; Bakker et al, 1998). In addition, MMRDX showed no substantial cardiotoxicity in rats (Danesi et al, 1993). Other anthracyclines showed less toxicity when administered as prolonged infusion than as bolus infusion (Legha et al, 1982*a*, 1982*b*). Therefore, we decided to perform a phase I study with MMRDX administered as a 3 h infusion every 4 weeks in order to try to increase the MTD as compared with bolus administration. In addition, we assessed pharmacokinetics of prolonged MMRDX-infusion.

*Received 6 January 1999*
*Revised 2 August 1999*
*Accepted 25 August 1999*

*Correspondence to:* EGE de Vries

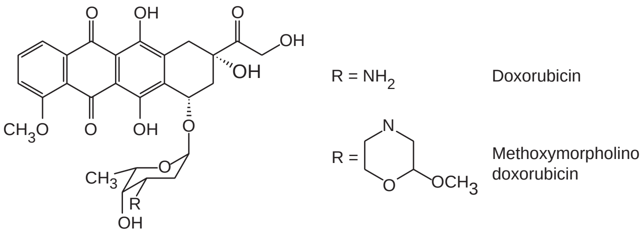

**Figure 1** Chemical structures of doxorubicin and methoxymorpholino doxorubicin

### PATIENTS AND METHODS

### Patients

The study was performed between December 1994 and June 1996. Patients with intrinsic anthracycline-resistant tumours were accrued in three different centres in Belgium and The Netherlands. Eligible were patients with histologically confirmed non-small-cell lung cancer (NSCLC), mesothelioma, head and neck, colorectal, renal, cervical cancer or adenocarcinoma of unknown origin, either metastatic or unresectable, not amenable to curative therapy. For colorectal cancer prior adjuvant chemotherapy ≥ 12 months or 5-fluorouracil (5-FU) treatment ≥ 6 weeks before study entry was allowed. For head and neck cancer prior chemotherapy as radiosensitization ≥ 6 weeks before study entry was allowed. Previous radiotherapy involving not more than 25% of bone marrow reserve was allowed but should have been completed for at least 4 weeks. Further inclusion criteria were an Eastern Cooperation Oncology Group performance status (PS ECOG) ≤ 2, life expectancy ≥ 3 months, neutrophils ≥ $2.0 \times 10^9$ $l^{-1}$, platelets ≥ $150 \times 10^9$ $1^{-1}$, creatinine ≤ 1.25 times the upper normal limit, and serum bilirubin, alkaline phosphatase, aspartate aminotransferase (ASAT) and alanine aminotransferase (ALAT) within the normal limits. In case of liver metastases patients were eligible if bilirubin was normal and liver enzymes were ≤ 2.5 times the upper normal limit. Excluded were patients with a history of prior malignancy (except for curatively treated carcinoma in situ of the cervix or localized epithelial skin cancer), an active infectious process, brain or leptomeningeal disease, a history of myocardial infarction within the last year, heart failure, arrythmias requiring permanent medication, or uncontrolled hypertension (≤ 200/110 mmHg). Pregnant or breast-feeding women, or fertile women refusing to use contraceptives or mentally incapacitated patients were also excluded. Pretreatment evaluation consisted of assessment of complete medical history, physical examination, ECG, measurement of LVEF by MUGA scan or echocardiography, and laboratory tests including complete blood count with differential, electrolytes, liver and renal function tests, total protein, albumen, glucose and urine analysis. The study was approved by all local medical ethics committees and all patients gave written informed consent.

### Study drug and dosing

MMRDX was obtained from Pharmacia & Upjohn (Milan, Italy) in freeze-dried vials containing 50 or 500 µg of product with lactose as excipient. Before administration, MMRDX was dissolved in 5 ml 0.9% sodium chloride to obtain concentrations of 10 and 100 µg $ml^{-1}$ respectively. The concentration of the administered drug was 30–50 µg $ml^{-1}$. MMRDX was administered by a continuous intravenous (i.v.) infusion during 3 h every 28 days for a maximum of six cycles. The starting dose was 1 mg $m^{-2}$ for the first three patients, and was extrapolated from the results obtained in the previous studies with bolus administration (Vasey et al, 1995; Bakker et al, 1998). If no DLT occurred, dose was escalated with 0.25 mg $m^{-2}$ for the next cohort of three patients. No intra-patient dose escalation was allowed. If DLT occurred in one out of three patients, another cohort of three patients was entered at the same dose level. If two or more patients showed DLT, further dose escalation was stopped. DLT was defined as National Cancer Institute Common Toxicity Criteria (CTC) grade IV complicated neutropenia, grade IV neutropenia lasting more than 8 days or grade III/IV thrombocytopenia. Other DLTs were CTC grade IV anaemia, grade ≥ III renal toxicity, grade ≥ III bilirubin, transaminases grade IV, or grade ≥ II at day 28, any combination of grade ≥ III clinical toxicities (except anorexia), grade ≥ I neurological toxicity, incomplete bone marrow recovery at day 42 or cardiotoxicity (defined as clinical signs of congestive heart failure or a decline in LVEF ≥ 15% to a value above the upper normal limit of the institution or ≥ 10% to a value below the lower normal limit). MTD was defined as the dose at which not more than than one out of three to six patients experienced DLT, with the next higher dose level causing DLT in two or more patients. Treatment delay up to 2 weeks for subsequent cycles of MMRDX was allowed if platelet count was still descending or < $150 \times 10^9$ $l^{-1}$, or if absolute granulocyte count was < $2.0 \times 10^9$ $l^{-1}$. Dose reduction by 10% at dose levels 1 and 1.25 mg $m^{-2}$ or by 0.25 mg $m^{-2}$ at dose level 1.5 mg $m^{-2}$ was performed if febrile neutropenia, any grade III/IV infection requiring i.v. antibiotics, absolute granulocyte count nadir < $0.5 \times 10^9$ $l^{-1}$ for > 8 days, platelets nadir < $50 \times 10^9$ $l^{-1}$, haemorrhagic diathesis occurred. If absolute granulocyte count at day 28 < $2.0 \times 10^9$ $l^{-1}$ or platelets at day 28 < $150 \times 10^9$ $l^{-1}$ occurred, but recovered after treatment delay dose reduction was also performed. Treatment was stopped after 2 weeks of treatment delay, if patients experienced unacceptable toxicity, or if patients showed progressive disease.

### Anti-emetics

Thirty minutes prior to MMRDX administration, patients received ondansetron 8 mg i.v. and dexamethasone 10 mg i.v. as anti-emetics. Thereafter, patients took orally ondansetron 2 × 8 mg at day 2 and 3, dexamethasone 2 × 9 mg at day 2 and dexamethasone 2 × 4.5 mg at day 3.

### Toxicity and response

Toxicity was evaluated weekly and graded according to CTC. Total blood count, white blood cell differential, and liver function tests were repeated weekly during treatment. A cycle was considered evaluable for haematological toxicity, if at least one haematological evaluation during the first 2 weeks and another evaluation between day 19 and 25 were performed. A cycle was evaluable for transaminases if at least one evaluation was performed between day 5 and 10, and for all other non-haematological toxicities if the assessment was performed within the end of cycle. Whenever a grade ≥ III toxicity occurred the cycle was always considered as

© 2000 Cancer Research Campaign evaluable. Physical examination and all laboratory tests except urine analysis were repeated once every cycle and after treatment. LVEF evaluation (either by MUGA-scan or echocardiography, but each patient being followed by the same method) was performed after every two cycles. Although tumour response was not an end point, tumour responses were assessed according to WHO criteria (World Health Organization, 1979) after the third and the last cycle.

### Pharmacokinetics

Only patients without liver metastases were enrolled in the pharmacokinetic part of the study. The pharmacokinetic profile of MMRDX was studied in plasma and urine obtained from patients in the first cycle during the first 120 h. All blood samples were collected in heparine-containing glass tubes and were protected from light because of photosensitivity of MMRDX. Blood samples were taken prior to the infusion of MMRDX, at 15, 30 min and 1.5 h during the infusion, at the end of infusion (3 h), and at 5, 15 and 30 min, and at 1, 2, 4, 6, 10, 24, 48, 72, 96 and 120 h thereafter. Samples were immediately centrifuged at 1200 ***g*** for 10 min at 4°C and plasma was stored in polypropylene tubes at –20°C until analysis. Determination of levels of MMRDX and its 13-dihydro metabolite (FCE 26176, 13-dihydro-3′-deamino-3′-(2(S)-methoxy-4-morpholinyl) doxorubicin) in plasma and urine was carried out by using high performance liquid chromatography with fluorescence detection by method of Breda et al (1992), with some modifications, as described by Bakker et al (1998). The detection limits for MMRDX and the 13-dihydro metabolite were 0.1 $\mu$g $l^{-1}$ in plasma and 0.5 $\mu$g $l^{-1}$ in urine.

### Data analysis

The plasma and urine pharmacokinetic parameters were calculated by standard non-compartmental analysis. Actual sampling times were used in the calculations. Pharmacodynamic analysis was performed by linear regression analysis between $C_{max}$, $AUC_{0\text{-}tz}$ and percentage change in haemoglobin, platelets, leucocytes and neutrophils during the first cycle.

### RESULTS

Fourteen male patients were entered in this study. Patient characteristics are shown in Table 1. Three patients were treated at dose level 1.0 mg $m^{-2}$ (8 cycles), six patients at 1.25 mg $m^{-2}$ (18 cycles) and five patients at 1.5 mg $m^{-2}$ (11 cycles). Seven patients did not receive any form of prior anticancer therapy. In five patients a potential risk factor for cardiac toxicity existed (mediastinal radiotherapy ($n$ = 1), hypertension ($n$ = 1), myocardial infarction and atrial fibrillation ($n$ = 1) and non-specific ST-T wave changes ($n$ = 2)). On therapy, at 1.5 mg $m^{-2}$, one patient died due to pulmonary embolism and one patient died due to sepsis during febrile neutropenia. In all other patients treatment was stopped for reason of disease progression. No tumour response was observed in 13 evaluable patients.

### Toxicity

Thirteen patients were evaluable for haematological toxicity. Table 2 shows CTC grade III and IV haematological toxicity at the different dose levels. No grade III/IV toxicity for haemoglobin was observed. At the lowest dose level no grade III/IV haematological toxicity occurred. Grade III/IV haematological toxicity was observed in one patient at 1.25 mg $m^{-2}$ MMRDX, and in all five patients at 1.5 mg $m^{-2}$. Grade IV neutropenia was the most common toxicity observed in 5/9 cycles. Three patients suffered from neutropenic fever and one of these patients died of septicaemia. Therefore, further dose escalation was stopped. Median nadir blood cell counts over all evaluable cycles are shown in Table 3. Nadirs occurred between day 15 and 29 for neutrophils and between day 8 and 28 for platelets over all dose levels. Four out of 11 cycles (three out of five patients) had to be reduced from 1.5 mg $m^{-2}$ to 1.25 mg $m^{-2}$ because of haematological toxicity. Overall, there was no cumulative haematological toxicity for subsequent cycles, except for platelets at the highest dose level.

Most common non-haematological toxicities were late nausea and vomiting, starting around 4 days after treatment. At 1 mg $m^{-2}$ non-haematological toxicity did not exceed CTC grade II. In patients treated with 1.25 mg $m^{-2}$ nausea, vomiting and diarrhoea exceeded grade II in one cycle, at 1.5 mg $m^{-2}$, nausea and vomiting exceeded grade II in two out of 11 cycles. Grade III/IV infection occurred in four out of 11 cycles and grade III/IV fatigue in one out of 11 cycles. At the end of treatment LVEF was evaluated in nine patients. No significant decreases in LVEF were observed in eight patients after six cycles ($n$ = 1), four cycles ($n$ = 1), three

**Table 1** Patient characteristics

| | |
|---|---|
| Number of patients | 14 |
| Median age in years (range) | 63 (41–76) |
| PS ECOG | |
| 0 | 7 |
| 1 | 6 |
| 2 | 1 |
| Diagnosis | |
| NSCLC | 4 |
| Renal cell cancer | 2 |
| Colorectal cancer | 4 |
| Head and neck cancer | 3 |
| Mesothelioma | 1 |
| Prior treatment | |
| Radiotherapy | 4 |
| Radio-, immuno- and chemotherapy | 2 |
| Chemo- and immunotherapy | 1 |
| Surgery | 4 |
| None | 3 |

Table 2 Percentage of total number of evaluable cycles with haematological toxicity

| | Dose level (mg $m^{-2}$) | Number of cycles | CTC grade toxicity (% of total number of cycles) III | IV |
|---|---|---|---|---|
| Leucocytes | 1.0 | 8 | 0 | 0 |
| | 1.25 | 17 | 6 | 0 |
| | 1.5 | 10 | 40 | 30 |
| Neutrophils | 1.0 | 7 | 0 | 0 |
| | 1.25 | 17 | 0 | 0 |
| | 1.5 | 7 | 29 | 43 |
| Platelets | 1.0 | 8 | 0 | 0 |
| | 1.25 | 17 | 0 | 0 |
| | 1.5 | 9 | 22 | 11 |

© 2000 Cancer Research Campaign

**Table 3** Median (range) nadir of blood cell counts over all evaluable cycles

| Dose level (mg m$^{-2}$) | Leucocytes ($\times 10^9$ l$^{-1}$) | Neutrophils ($\times 10^9$ l$^{-1}$) | Platelets ($\times 10^9$ 1$^{-1}$) |
|---|---|---|---|
| 1.0 | 6.0 (3.9–15.1) | 3.3 (2.4–12.7) | 197 (130–356) |
| 1.25 | 2.7 (1.0–10.4) | 1.9 (0.5–8.3) | 190 (60–328) |
| 1.5 | 1.4 (0.2–7.5) | 0.5 (0.2–7.3) | 55 (8–119) |

cycles ($n$ = 4) and after two cycles ($n$ = 2). One patient at the highest dose level showed a decrease in LVEF of 13% after three cycles, but LVEF remained in the normal range. In five patients LVEF was not evaluated (after three cycles ($n$ = 2) and after one cycle ($n$ = 3)), but no clinical signs of heart failure were observed. Transient elevations in transaminases (ALAT and ASAT) were observed at all dose levels. Maximum toxicity for transaminases reached CTC grade III at dose level 1.25 mg m$^{-2}$ in 2/16 cycles and at 1.5 mg m$^{-2}$ in 4/11 cycles. Transient elevations in total bilirubin were observed at 1.25 and 1.5 mg m$^{-2}$ reaching CTC grade II in 2/15 and 3/11 cycles respectively. No phlebitis at the infusion site and no nephro- or neurotoxicity was observed.

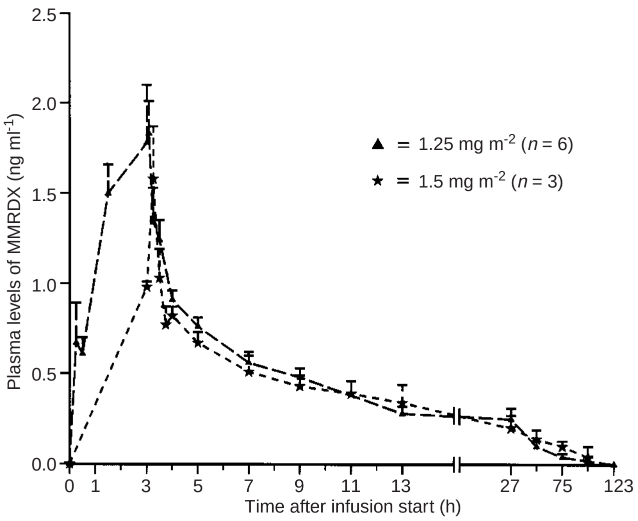

**Figure 2** Mean plasma levels of MMRDX at dose levels 1.25 mg m$^{-2}$ and 1.5 mg m$^{-2}$. Error bars indicate standard error of the mean

### Pharmacokinetics

Plasma samples of 12 patients were available for non-compartmental pharmacokinetic analysis. Mean plasma levels of MMRDX at 1.25 mg m$^{-2}$ and 1.5 mg m$^{-2}$ are shown in Figure 2. Pharmacokinetics parameters are shown in Table 4. In five patients only $C_{max}$, $AUC_{0\text{-}tz}$, Ae and percentage of dose excreted could be calculated due to missing samples. Differences in median $C_{max}$ between the dose levels were statistically not significant. At 1 mg m$^{-2}$, one patient showed a high $C_{max}$ of 6.17 ng ml$^{-1}$, all other patients showed $C_{max}$ around 2.0 ng ml$^{-1}$ for all dose levels. However, $AUC_{0\text{-}tz}$ increased with the dose. $AUC_{0\text{-}\infty}$ calculated from non-compartmental analysis was around 30 ng h ml$^{-1}$ and similar for all dose levels, based on data obtained from seven patients. At all dose levels a rather long $t_{\frac{1}{2}z}$, a large $V_{ss}$, and a rapid plasma clearance was observed. Urine excretion (Ae) of MMRDX was very low, up to 2.5% of the administered dose. Also urine excretion of the 13-dihydro metabolite of MMRDX was low, up to 2.3% of the administered dose. Pharmacodynamic analysis revealed no correlation between $AUC_{0\text{-}tz}$ or $C_{max}$ and nadirs of haemoglobin, platelets, leucocytes and neutrophils.

### DISCUSSION

This study shows that prolonged infusion of 1.5 mg m$^{-2}$ MMRDX shows more toxicity than observed after bolus infusion (Vasey et al, 1995; Bakker et al, 1998). MTD was lowered to 1.25 mg m$^{-2}$. Late haematological toxicity around day 22 was the main toxicity. Dose-limiting CTC grade IV neutropenia was observed in 3/7 (43%) evaluable cycles at 1.5 mg m$^{-2}$, with one septic death. Previous studies with the same dose MMRDX as bolus infusion every 3 weeks (Vasey et al, 1995) and every 4 weeks (Bakker et al, 1998) showed grade IV neutropenia in 14% and 9% of administered cycles respectively. Also grade III/IV thrombocytopenia occurred more frequently after prolonged infusion of 1.5 mg m$^{-2}$ MMRDX than after bolus infusion. This unexpected increase in haematological toxicity by prolonging the infusion indicates that MMRDX should be administered as bolus infusion.

Non-haematological toxicity was comparable to the earlier studies with MMRDX and consisted mainly of nausea and vomiting starting 4 days after treatment, hepatic toxicity and to a lesser extent mucositis and fatigue. No cardiotoxicity was observed in the present study, although follow-up was relatively

**Table 4** Pharmacokinetics parameters (median (range)) as obtained by non-compartmental analysis

| | Dose level in mg m$^{-2}$ (number of patients) | | |
|---|---|---|---|
| | 1.0 ($n$ = 3) | 1.25 ($n$ = 6) | 1.5 ($n$ = 3) |
| $C_{max}$ (ng ml$^{-1}$) | 2.8 (1.8–6.2) | 2.3 (1.1–2.7) | 1.4 (1.2–2.1) |
| AUC (ng h ml$^{-1}$) 0-$t_z$ | 9.4 (8.3–18.3) | 15.1 (10.1–30.4) | 21.4 (5.7–26.9) |
| Cl (ml min$^{-1}$ m$^{-2}$) | 540.5[a] | 649.0 (506.3–812.3)[c] | 741.2 (659–823.2)[b] |
| $t_{\frac{1}{2}z}$ (h) | 68.6[a] | 60.9 (45.6–88.8)[c] | 46.5 (36.1–56.9)[b] |
| $V_z$ (l m$^{-2}$) | 3209[a] | 3942.5 (1997–4540)[c] | 2908.5 (2574–3243)[b] |
| $V_{ss}$ (l m$^{-2}$ | 2502[a] | 2891.5 (1742–3371)[c] | 2693.0 (2585–2801)[b] |
| Ae in urine 0–72 h (µg) | 28.4 (1.6–34.7) | 52.7 (6.7–68.0) | 69.3 (49.0–73.6) |
| % Dose in urine | 1.6 (0.53–1.74) | 2.1 (0.35–2.6) | 2.47 (1.71–2.49) |

[a]$n$ = 1; [b]$n$ = 2; [c]$n$ = 4.

© 2000 Cancer Research Campaign short. Also in the previous studies no cardiotoxicity was observed, except for two patients in the phase II study who had other risk factors as well.

In the past, pharmacodynamic analysis has revealed correlations between AUC and haematological toxicity for epirubicin and doxorubicin (Jakobsen et al, 1991; Piscitelli et al, 1993). For MMRDX, we could not find a correlation between AUC or $C_{max}$ and haematological toxicity. Also, Bakker et al (1998) could not establish a correlation between $C_{max}$, AUC or levels of MMRDX in leucocytes and haematological or non-haematological toxicity. Also, the calculated pharmacokinetics parameters in the present study were similar to those obtained from bolus administration in earlier investigations. In our study $AUC_{0-\infty}$ was just slightly higher compared to bolus infusion (Bakker et al, 1998), while $AUC_{0-\infty}$ in the phase I study (Vasey et al, 1995) was higher than the $AUC_{0-\infty}$ in our study. Therefore, we conclude that these parameters reveal no explanation for the increased haematological toxicity. Further pharmacokinetics parameters were in reasonable agreement with earlier data. The phase I study (Vasey et al, 1995) showed a $t_{\frac{1}{2}}$ for MMRDX of 40 h after a rapid distribution phase. Plasma clearance was 650 ml $min^{-1}$ $m^{-2}$. The phase II study (Bakker et al, 1998) showed a $t_{\frac{1}{2}}$ of the elimination phase of 49 h, a plasma clearance of 620 ml $min^{-1}$ $m^{-2}$. This study also showed that leucocyte levels of MMRDX were 400- to 600-fold higher than plasma levels. This, together with the large $V_{ss}$, long $t_{\frac{1}{2}}$, low renal excretion and a rapid clearance from the circulation, indicates that MMRDX is rapidly distributed into tissues, which is not surprising since MMRDX is a highly lipophilic drug (Acton et al, 1984; Streeter et al, 1986). Therefore, tissue levels might be more predictive for toxicity than plasma levels or $C_{max}$ and AUC obtained from plasma.

In the present study no tumour responses to MMRDX were observed. Vasey and co-workers (Vasey et al, 1995) reported four responses in head and neck (one out of three), cervical cancer (one out of five), and colorectal cancer (two out of 20). The phase II study (Bakker et al, 1998) showed one partial response in one out of 17 NSCLC.

In conclusion, prolonged administration of MMRDX shows more myelosuppression than bolus infusion with neutropenia as DLT. Pharmacokinetics parameters did not explain this increase in toxicity. Non-haematological toxicity was similar. No clear signs of cardiotoxicity have been observed, although follow-up was relatively short.

### ACKNOWLEDGEMENTS

Methoxymorpholino doxorubicin was supplied by Pharmacia & Upjohn, Nerviano, Italy, who also supported data management.

### REFERENCES

Acton EM, Tong GL, Mosher CW and Wolgemuth RL (1984) Intensely potent morpholinyl anthracyclines. *J Med Chem* **27**: 638–645

Bakker M, Renes J, Groenhuijzen A, Visser P, Timmer-Bosscha H, Muller M, Groen HJM, Smit EF and de Vries EGE (1997) Mechanisms for high methoxymorpholino doxorubicin cytotoxicity in doxorubicin-resistant tumor cell lines. *Int J Cancer* **73**: 362–366

Bakker M, Droz JP, Hanauske AR, Verweij J, van Oosterom AT, Groen HJM, Pacciarini MA, Domenigoni L, van Weissenbruch F, Pianezzola E and de Vries EGE (1998) Broad phase II and pharmacokinetic study of methoxymorpholino doxorubicin (FCE23762-MMRDX) in NSCLC, renal cancer and other solid tumour patients. *Br J Cancer* **77**: 139–146

Breda M, Pianezzola E and Benedetti MS (1992) Determination of 3′-deamino-3′-[2(S)-methoxy-4-morpholinyl]doxorubicin, a new morpholinyl anthracycline, in plasma by high-performance liquid chromatography with fluorescence detection. *J Chromatogr* **578**: 309–315

Danesi R, Agen C, Grandi M, Nardini V, Bevilacqua G and Del Tacca M (1993) 3′-Deamino-3′-(2-methoxy-4-morpholinyl)-doxorubicin (FCE 23762): a new anthracycline derivative with enhanced cytotoxicity and reduced cardiotoxicity. *Eur J Cancer* **29A**: 1560–1565

de Jong S, Zijlstra JG, de Vries EGE and Mulder NH (1990) Reduced DNA topoisomerase II activity and drug-induced DNA cleavage activity in an adriamycin-resistant human small cell lung carcinoma cell line. *Cancer Res* **50**: 304–309

Deffie AM, Batra JK and Goldenberg GJ (1989) Direct correlation between DNA topoisomerase II activity and cytotoxicity in adriamycin-sensitive and -resistant P388 leukemia cell lines. *Cancer Res* **49**: 58–62

Ford JM and Hait WN (1990) Pharmacology of drugs that alter multidrug resistance in cancer. *Pharmacol Rev* **42**: 155–199

Jakobsen P, Bastholt L, Dalmark M, Pfeiffer P, Petersen D, Gjedde SB, Sandberg E, Rose C, Nielsen OS and Mouridsen HT (1991) A randomized study of epirubicin at four different dose levels in advanced breast cancer. Feasibility of myelotoxic prediction through single blood-sample measurement. *Cancer Chemother Pharmacol* **28**: 465–469

Johnston JB and Glazer RI (1983) Cellular pharmacology of 3′-(4-morpholinyl) and 3′-(4-methoxy-1-piperidinyl) derivatives of 3′-deaminodaunorubicin in human colon carcinoma cells in vitro. *Cancer Res* **43**: 1606–1610

Kaye S and Merry S (1985) Tumour cell resistance to anthracyclines: a review. *Cancer Chemother Pharmacol* **14**: 96–103

Kuhl JS, Duran GE, Chao NJ and Sikic BI (1993) Effects of the methoxymorpholino derivative of doxorubicin and its bioactivated from versus doxorubicin on human leukemia and lymphoma cell lines and normal bone marrow. *Cancer Chemother Pharmacol* **33**: 10–16

Lau DH, Duran GE, Lewis AD and Sikic BI (1994) Metabolic conversion of methoxymorpholinyl doxorubicin: from a DNA strand breaker to a DNA cross-linker. *Br J Cancer* **70**: 79–84

Legha SS, Benjamin RS, Mackay B, Ewer M, Wallace S, Valdivieso M, Rasmussen SL, Blumenschein GR and Freireich EJ (1982a) Reduction of doxorubicin cardiotoxicity by prolonged continuous intravenous infusion. *Ann Intern Med* **96**: 133–139

Legha SS, Benjamin RS, Mackay B, Yap HY, Wallace S, Ewer M, Blumenschein GR and Freireich EJ (1982b) Adriamycin therapy by continuous intravenous infusion in patients with metastatic breast cancer. *Cancer* **49**: 1762–1766

Piscitelli SC, Rodvold KA, Rushing DA and Tewksbury DA (1993) Pharmacokinetics and pharmacodynamics of doxorubicin in patients with small cell lung cancer. *Clin Pharmacol Ther* **53**: 555–561

Ripamonti M, Pezzoni G, Pesenti E, Pastori A, Farao M, Bargiotti A, Suarato A, Spreafico F and Grandi M (1992) In vivo anti-tumour activity of FCE23762, a methoxymorpholinyl derivative of doxorubicin active on doxorubicin-resistant tumour cells. *Br J Cancer* **65**: 703–707

Roninson IB (1997) The role of the mdr1 (P-glycoprotein) gene in multidrug resistance in vitro and in vivo. *Biochem Pharmacol* **43**: 95–102

Streeter DG, Johl JS, Gordon GR and Peters JH (1986) Uptake and retention of morpholinyl anthracyclines by adriamycin-sensitive and -resistant P388 cells. *Cancer Chemother Pharmacol* **16**: 247–252

van der Graaf WTA, Mulder NH, Meijer C and de Vries EGE (1995) The role of methoxymorpholino anthracycline and cyanomorpholino anthracycline in a sensitive small-cell lung-cancer cell line and its multidrug-resistant but P-glycoprotein-negative and cisplatin-resistant counterparts. *Cancer Chemother Pharmacol* **35**: 345–348

Vasey PA, Bissett D, Strolin Benedetti M, Poggesi I, Breda M, Adams L, Wilson P, Pacciarini MA, Kaye SB and Cassidy J (1995) Phase I clinical and pharmacokinetic study of 3′-deamino-3′-(2-methoxy-4-morpholinyl)-doxorubicin (FCE23762). *Cancer Res* **55**: 2090–2096

Wassermann K, Markovits J, Jaxel C, Capranico G, Kohn KW and Pommier Y (1990) Effects of morpholinyl doxorubicins, doxorubicin, and actinomycin D on mammalian DNA topoisomerase I and II. *Mol Pharmacol* **38**: 38

Wassermann K, Newman RA, Davis FM, Mullins TD and Rose KM (1988) Selective inhibition of human ribosomal gene transcription by the morpholinyl anthracyclines cyanomorpholinyl- and morpholinyldoxorubicin. *Cancer Res* **48**: 4101–4106

World Health Organization (1979) *Handbook for Reporting Results of Cancer Treatment*. WHO offset publication no. 48. Nijhoff: Den Haag, The Netherlands

*© 2000 Cancer Research Campaign*
